# Why do men have worse COVID-19-related outcomes? A systematic review and meta-analysis with sex adjusted for age

**DOI:** 10.1590/1414-431X2021e11711

**Published:** 2022-02-16

**Authors:** J. Fabião, B. Sassi, E.F. Pedrollo, F. Gerchman, C.K. Kramer, C.B. Leitão, L.C. Pinto

**Affiliations:** 1 Divisão de Medicina Interna, Hospital Nossa Senhora da Conceição, Porto Alegre, RS, Brasil; 2 Programa de Pós-Graduação em Ciências Médicas: Endocrinologia, Divisão de Endocrinologia, Hospital de Clínicas de Porto Alegre, Universidade Federal do Rio Grande do Sul, Porto Alegre, RS, Brasil; 3 Mount Sinai Hospital, University of Toronto, Toronto, Ontario, Canada

**Keywords:** COVID-19, Sexual hormone, Meta-analysis

## Abstract

We aimed to study the mechanism behind worse coronavirus disease-19 (COVID-19) outcomes in men and whether the differences between sexes regarding mortality as well as disease severity are influenced by sex hormones. To do so, we used age as a covariate in the meta-regression and subgroup analyses. This was a systematic search and meta-analysis of observational cohorts reporting COVID-19 outcomes. The PubMed (Medline) and Cochrane Library databases were searched. The primary outcome was COVID-19-associated mortality and the secondary outcome was COVID-19 severity. The study was registered at PROSPERO: 42020182924. For mortality, men had a relative risk of 1.36 (95%CI: 1.17 to 1.59; *I^2^* 63%, P for heterogeneity <0.01) compared to women. Age was not a significant covariate in meta-analysis heterogeneity (P=0.393) or subgroup analysis. For disease severity, being male was associated with a relative risk of 1.29 (95%CI: 1.19 to 1.40; *I^2^* 48%, P for heterogeneity <0.01) compared to the relative risk of women. Again, age did not influence the outcomes of the meta-regression (P=0.914) or subgroup analysis. Men had a higher risk of COVID-19 mortality and severity regardless of age, decreasing the odds of hormonal influences in the described outcomes.

## Introduction

In late 2019, the world was introduced to a new virus that was first described in the city of Wuhan, China. This virus was later named severe acute respiratory syndrome coronavirus 2 (SARS-CoV-2), and the related clinical syndrome was named coronavirus disease 2019 (COVID-19). Soon, COVID-19 spread worldwide and reached pandemic proportions in March 2020 (1). It was estimated that by September 2021, more than 219 million people around the globe were infected by COVID-19, with more than 4 million deaths.

Several reports have identified risk factors for disease severity and mortality, especially older age, hypertension, diabetes, obesity, and cardiovascular disease. Nonetheless, the same reports showed an increased risk of complications in men (2). Notably, among the nine deaths initially reported in Italy in patients younger than 40 years, eight were men (2,3). The difference in clinical presentation between men and women may be directly mediated by sex hormones (4). As testosterone physiologically decreases in older males (5), age may play an important role in mediating adverse COVID-19 outcomes according to sex. In this sense, differences in COVID-19 severity and mortality between men and women would be greater in younger patients and the magnitude of the association would be weakened as age increases, following the predicted decrease in testosterone production in older people. With this in mind, we aimed to assess the differences between the sexes regarding mortality as well as severity of COVID-19, taking into account age as a covariate, evaluated by meta-regression and subgroup analyses.

## Material and Methods

We performed a systematic search and meta-analysis of observational cohorts reporting COVID-19 outcomes, which included detailed descriptions of patients' clinical profiles and disease severity (according to authors' definition: peripheral oxygen saturation of <90%, need for intensive care unit admission, and/or need for mechanical ventilation) and/or mortality. The PubMed (Medline) and Cochrane Library databases were searched up to May 13th, 2021. The primary outcome was COVID-19-associated mortality and the secondary outcome was COVID-19 severity. This systematic review and meta-analysis was registered in PROSPERO (registration number 42020182924).

The collected data included the author's name, year of publication, country of publication, and ages of the included participants, as well as the difference in age between groups, number of patients with severe COVID-19, number of patients with mild/moderate COVID-19, number of deaths due to COVID-19, and number of survivors. Data were extracted by two researchers (J.F. and B.S.), disagreements were solved by a third party (L.C.P.) and the quality of the analyzed studies was assessed using the Meta-Analyses of Observational Studies in Epidemiology (MOOSE) Guidelines (6).

We performed direct meta-analyses and calculated the relative risk for both outcomes (COVID-19 mortality and severity). To analyze the influence of age on the outcomes (as a surrogate variable for hormonal reserve), we performed meta-regression using the mean age of study participants as a covariate. Furthermore, subgroup meta-analyses according to study participants' mean age (below 50 years, 51-60 years, and above 60 years) were planned. Heterogeneity was assessed by the Cochran Q test (a P value of 0.1 was considered statistically significant) and the *I* ^2^ test (values greater than 50% were considered to indicate elevated statistical heterogeneity). Publication bias was assessed by funnel plot analysis. Analyses were performed using R software version 4.0.3 “Bunny-Wunnies Freak Out” (The R Foundation for Statistical Computing).

## Results

**Figure 1:**
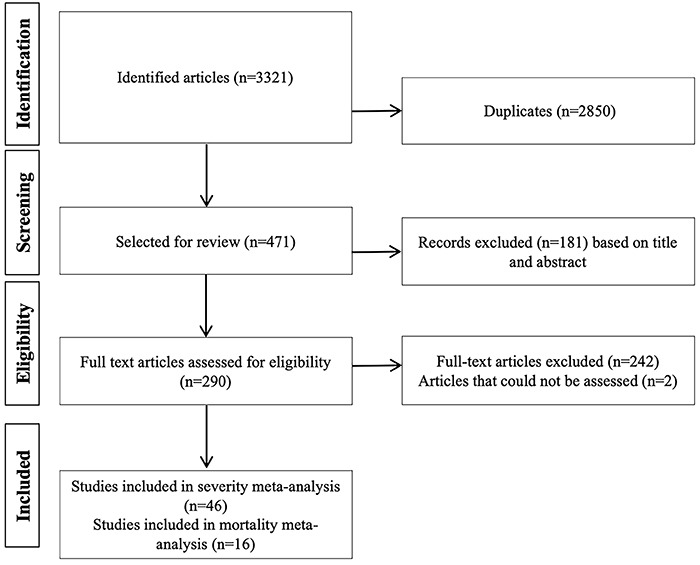
Study flowchart.

The initial search retrieved 3321 articles. Six hundred ninety-six articles were published prior to 2019 and were excluded. Of the remaining articles, 16 studies had information about patient sex and COVID-19-associated mortality (20,601 patients) and 46 had information on disease severity (12,513 patients) (Figure 1).

**Figure 2:**
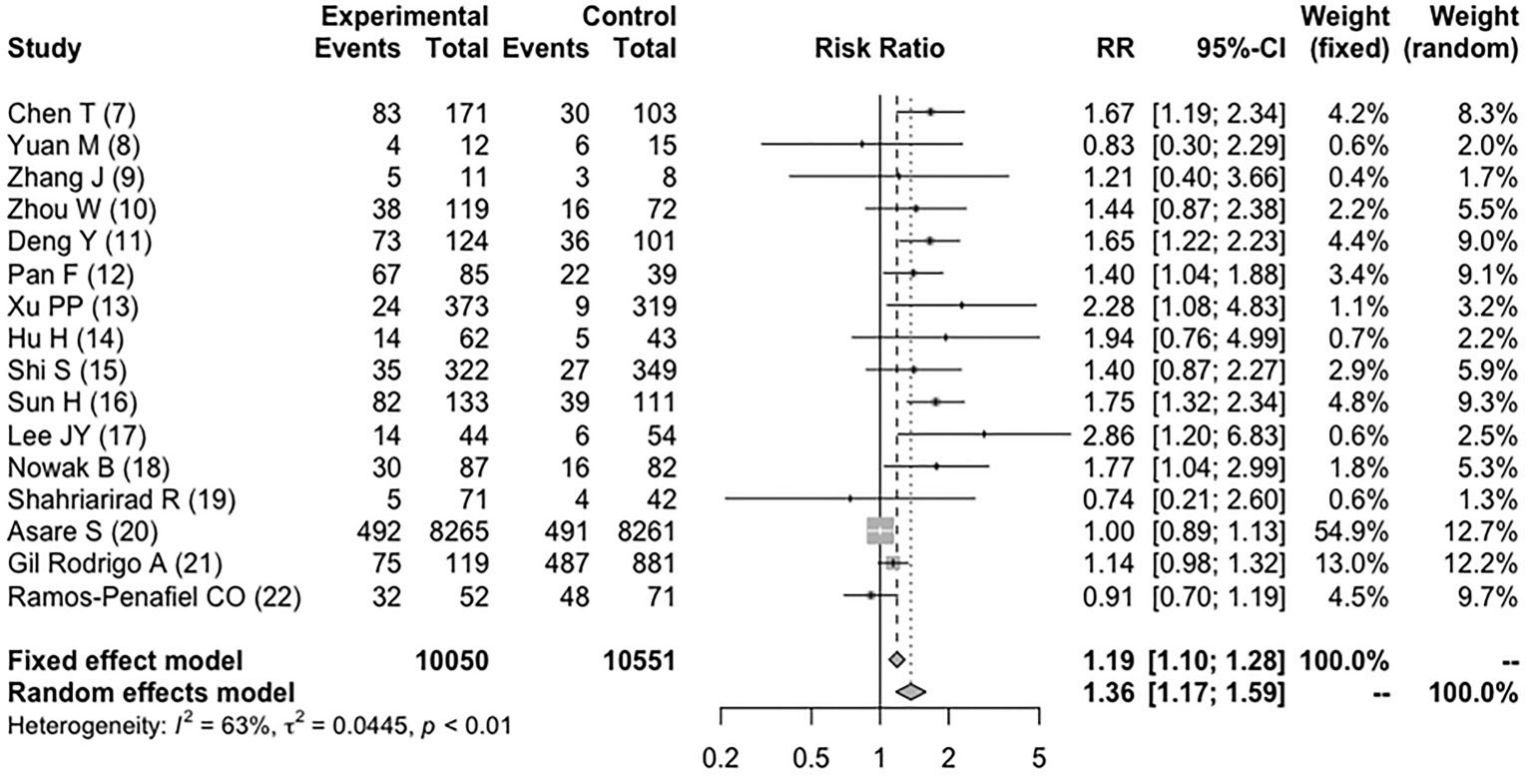
Forest plot for COVID-19 mortality in male patients.

Men had a relative risk of mortality of 1.36 (95%CI: 1.17 to 1.59; *I^2^* 63%, P for heterogeneity <0.01, Figure 2; 7-22) compared with women. Age did not affect the outcomes in the meta-regression (P=0.393). The subgroup analysis was performed with studies pooled into two groups (cutoff of 60 years) because only one study with a mean participant age below 50 years was identified. Men younger than 60 years had a RR of 1.53 (95%CI: 0.96 to 2.43; *I^2^* 18%, P for heterogeneity 0.29; 4 studies), while those above 60 years of age had a RR of 1.54 (95%CI: 1.29 to 1.83; *I^2^* 0%, P for heterogeneity 0.67; 9 studies). The RR for mortality in men younger than 60 years was not significant. However, the magnitude of the association was similar to that found in older subjects (1.53 *vs* 1.54), and this particular meta-analysis was based only on four studies (10,13,19,22), which probably lacks power to reach definitive conclusions in this subgroup.

**Figure 3:**
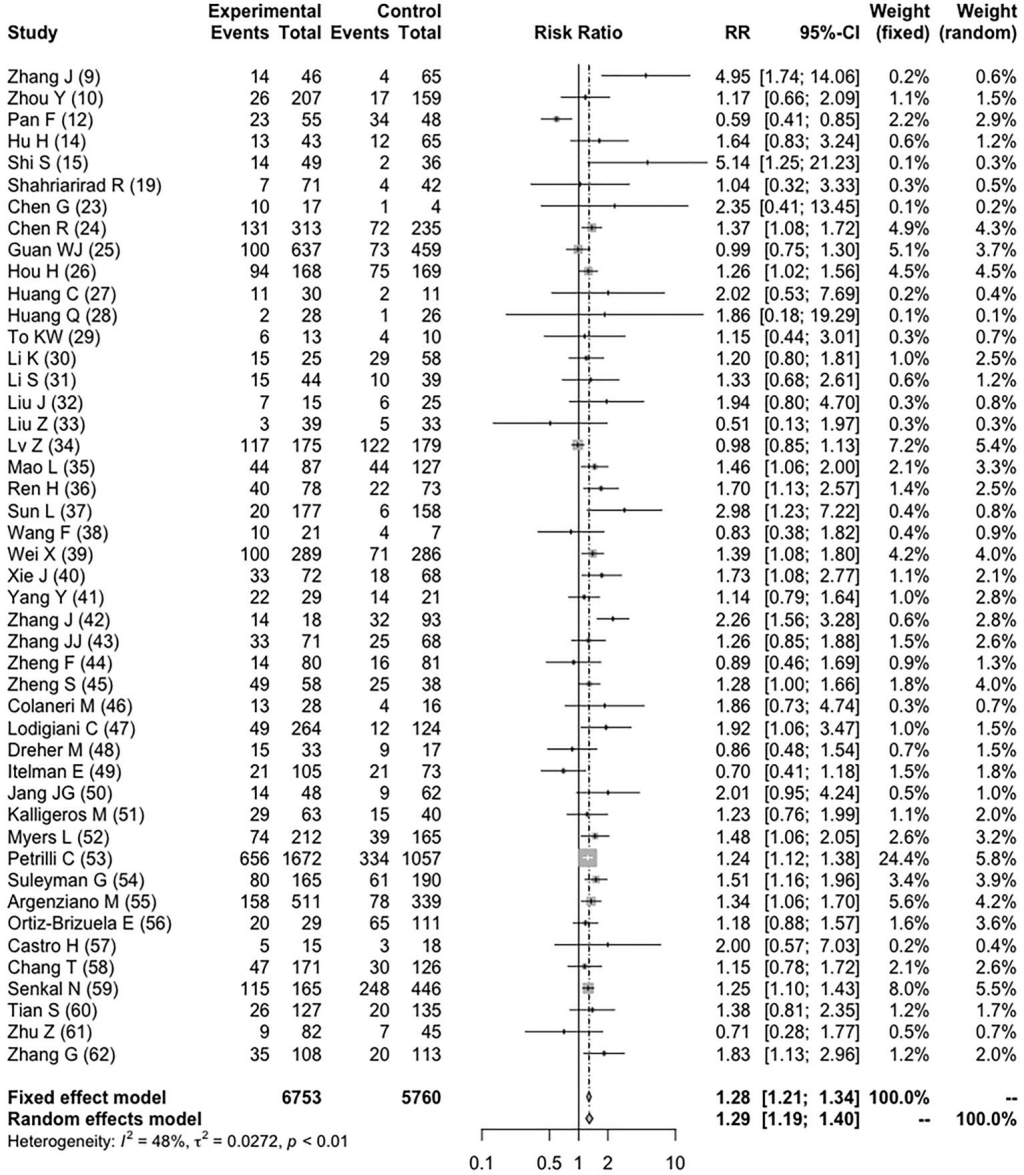
Forest plot for COVID-19 severity in male patients.

For disease severity, being male was associated with a relative risk of 1.29 (95%CI: 1.19 to 1.40; *I^2^* 48%, P for heterogeneity <0.01) compared to females (Figure 3; 7-22) and S2 (9,10,12,14,15,19,23-62). Age again was not a significant covariate implicated in meta-analysis heterogeneity identified by meta-regression (p=0.914). The subgroup analysis included studies classified based on patients' mean age in three strata: below 50 years, 51-60 years, and above 60 years. In this analysis, the magnitude of the association of male sex and COVID-19 severity was similar regardless of age group: the group of patients <50 years old had an RR of 1.37 (95%CI: 1.01 to 1.86; *I^2^* 44%, P for heterogeneity 0.06; 11 studies), the group of patients aged 51-60 years had an RR of 1.32 (95%CI: 1.11 to 1.56; *I^2^* 57%, P for heterogeneity <0.01, 18 studies), and the group of patients aged >60 years had a RR of 1.24 (95%CI: 1.12 to 1.38; *I^2^* 37%, P for heterogeneity 0.08, 14 studies). The quality assessment of the included studies is presented in Supplementary Tables S1 (7-22) and S2 (9,10,12,14,15,19,23-62), showing an overall good quality, and no publication bias was identified after inspection of the funnel plot (Supplementary Figure S1).

## Discussion

This systematic review and meta-analysis confirmed that COVID-19 severity and mortality are increased in men. Age was not an important cofactor modulating these interactions, as associations were maintained at similar levels across all age strata.

The higher rate of mortality in men with COVID-19 may be explained by several factors. There are some differences between male and female detection of nucleic acids by innate immunity, with pattern recognition receptors (PRRs) differing between the sexes (63). The Toll-like receptor 7 (TLR7) gene may escape X inactivation, resulting in higher expression levels of TLR7 in females than in males (64). Moreover, *in vitro* experiments have shown that the exposure of peripheral blood mononuclear cells (PBMCs) to TLR7 ligands causes higher production of interferon–α (IFN-α) in cells in women than in men, and plasmacytoid dendritic cells (pDCs) from female humans have higher basal levels of IFN regulatory factor 5 (IRF5) and IFN-α production following TLR7 ligand stimulation (65). A review that examined the association between sex differences in the immune system concluded that sex-based immunological differences contribute to variations in susceptibility to infectious diseases. For example, sex differences in human leukocyte antigen (HLA) alleles and genes that encode for the interleukin (IL) receptors IL-4, IL-10, and IL-12 have each been associated with differential antibody responses to several vaccines in children and adults. In those cases, hormonal mechanisms are hypothesized to be implicated (66). Another review documented that women, especially during the reproductive years, are at increased risk of developing autoimmune diseases but are more resistant to infections than men, which might be mediated by several factors, including sex hormones (66,67). The concept of sex-based immunological differences driven by sex hormones and the X chromosome has been well described by Elgendy et al. (68). Angiotensin-converting enzyme-2 (ACE2) encoded by the ACE 2 gene has been proven to be the receptor for both SARS coronavirus (SARS-CoV) and human respiratory coronavirus NL63 (69). Different studies have quantified the expression of ACE 2 proteins in human cells based on sex and ethnicity. The expression level and pattern of human ACE 2 using single-cell RNA sequencing (RNA-seq) indicates that Asian males have higher expression of ACE 2 than females (70). 

Interestingly, the blockage of estrogen receptors increased mortality from SARS-CoV infection in female mice, suggesting a role for estrogen receptors in modulating responses to viral infections. Similarly, theories regarding a benefit of testosterone blockage have been postulated (70,71). Based on this interesting but unproven hypothesis, clinical trials are recruiting volunteers to test the effect of lowering testosterone production or action in men infected by SARS-CoV-2. Clinical trials are testing two anti-androgen medications: degarelix acetate (an LHRH analog that decreases pituitary gonadotrophin release and ultimately testosterone production) (72) and dutasteride (a 5-alpha reductase inhibitor that blocks the enzyme that converts testosterone to a more potent androgen, dihydrotestosterone) (73). Additionally, results from a controversial study analyzing the effect of proxalutamide (an androgen receptor antagonist) have been recently published (74).

However, our meta-analysis results do not support the “hormonal theory”. If higher testosterone values were the sole factor responsible for sex differences in COVID-19 outcomes, a decrease in the magnitude of the association would be expected as patient age increased. Additionally, if estrogen were a protective factor, the “sex gap” would decrease with older age, as it seems to for cardiovascular disease incidence among men and women (75). This phenomenon was not observed in our study. In our analysis, the magnitude of the association between COVID-19 outcomes and male sex was unchanged and not modulated by age.

Our findings may have two explanations. First, the similar RR for disease severity observed in younger (presumably, with higher testosterone production) and older men (with lower testosterone production) may reflect different factors determining outcomes across ages. In young healthy men, testosterone may have implications in disease severity, while in older men, other factors, such as the increased prevalence of obesity, diabetes, hypertension, and cardiovascular disease, surpass the possible protection of lower testosterone production. Second and most likely, other theories, in addition to testosterone effects, would explain the worse outcomes observed in men. Different outcomes for men and women might be due to health behaviors, as men are more prone to smoke and be heavy drinkers than women (76). Additionally, other risk factors for COVID-19 severity, such as hypertension and cardiovascular disease, are more prevalent in men. Furthermore, a study conducted in Spain reported that women had more responsible attitudes toward the COVID-19 pandemic than men (77). Some shortcomings of the current analysis should be highlighted. Given the observational nature of the included studies and the pooled analysis that characterizes a meta-analysis, it was not possible to directly assess the effects of hormonal values or to isolate age from sex at the individual level. A direct assessment would only be possible if we had access to individual data to perform meta-analysis on data at the individual level. Additionally, the number of studies reporting mortality was low, precluding sufficient power to properly conduct the meta-regression.

In conclusion, this meta-analysis, unlike others (78,79), assessed the possible interaction among sex, age, and COVID-19 mortality and severity. Men are at higher risk for COVID-19 mortality and severe cases regardless of age, decreasing the odds for hormonal influences in the described outcomes. Further studies, including a meta-analysis with data at the patient level, should be conducted to clarify the mechanisms for poorer outcomes among men with COVID-19.

## Supplementary Material

Click here to view [pdf].
